# Heating and Strain Sensing Elements Based on Segregated Polyethylene/Carbon Black Composites in Polymer Welded Joints

**DOI:** 10.3390/ma17153776

**Published:** 2024-08-01

**Authors:** Yevheniia Buinova, Anastasiia Kobyliukh, Yevgen Mamunya, Oleksii Maruzhenko, Mykola Korab, Barbara Trzebicka, Urszula Szeluga, Marcin Godzierz

**Affiliations:** 1 Center of Polymer and Carbon Materials, Polish Academy of Sciences, 34. M. Curie-Skłodowskiej St., 41-800 Zabrze, Poland; jennushaa@gmail.com (Y.B.); akobyliukh@cmpw-pan.pl (A.K.); uszeluga@cmpw-pan.pl (U.S.); 2 E.O. Paton Electric Welding Institute of the National Academy of Sciences of Ukraine, 11. Kazymyr Malevych St., 03680 Kyiv, Ukraine; ymamunya@ukr.net (Y.M.); a.v.maruzhenko@gmail.com (O.M.); korab_nikolay@ukr.net (M.K.)

**Keywords:** conductive polymer matrix composites, carbon fillers, segregated structure, strain sensing, electrofusion welding

## Abstract

The development of easy and direct real-time monitoring of welded joint quality instead of surface damage analysis is crucial to improve the quality of industrial products. This work presents the results of high-density polyethylene (HDPE)-based composites with various carbon black (CB) content (from 20 to 30 vol.%) for use as a heating element and strain sensor in electrofusion-welded polymer joints. The pyroresistive heating process was used to determine the effect of generated Joule heat during welding on the structure and sensor properties of polymer–carbon composites. It is shown that the generation of Joule heat depends on the nanocarbon content and affects the crystallinity of the polymer matrix. The partial disruption of the conductive path of carbon black particles was observed and, as a result, a decrease in electrical conductivity for composites with lower CB content after welding was found. For the highest CB amount, conductivity increased, which is caused by smaller particle-to-particle distance for filler paths. Therefore, the best balance between pyroresistive and sensor properties was found.

## 1. Introduction

The quality assurance of polymeric products is a critical aspect of industrial manufacturing and can be achieved by real-time monitoring of the production process and nondestructive testing (NDT) techniques of the final products. Nondestructive testing is a method of evaluation of material integrity regarding the presence of surface or internal defects and/or the quality of product parts and their joint connections, in particular, welded joints, with no effect on the material’s usability [1].

Simple products based on pure polymers or polymer composites can be formed using well-known technologies, such as injection molding or hot pressing. However, for products with more complicated shapes, it is necessary to use technologies, including welding, to join the individual parts made by the abovementioned methods [2]. 

In the industrial process, welding of thermoplastic polymers is performed [3] using several different plastic welding techniques, and the choice of weld depends on the polymer type, its physical and chemical properties, and the form of the product [4,5,6,7]. The methods of welding polymer materials can include, among others, ultrasonic welding, friction welding, vibration welding, laser welding, induction welding, high-frequency welding, resistance welding, solvent welding, hot-air welding and hot-plate welding [8]. For most polymer welding techniques, a high equipment investment is required while welding strength is not as high.

One of the most frequently used welding technologies is electrofusion welding. This technique is based on a welding process based on Joule heat release of the embedded thermal (thermally active) element and heated surfaces of the joined details to a temperature higher than their melting range. These built-up elements of different geometry can be obtained from metal mesh or electrically conductive polymer composites (CPCs) [2,9].

Extensive research and development of polymer composites with different fillers, including carbon fillers (carbon black, graphite, carbon fibers, and carbon nanotubes) [7,10], make it possible to formulate many different conductive polymer materials based on a nonconductive typical polymer matrix, i.e., polyolefins, polyamides, polyesters, epoxy resins, PEEK, and others [11].

The application of these fillers to the polymer matrix results in the final conductive composites according to the percolation theory [12], i.e., a conductive framework is formed in the polymer volume at a specific filler concentration that exceeds the critical concentration (φ_c_), named the percolation threshold, when a significant increase in conductivity of several orders of magnitude is observed. The type of filler used, as well as the process of producing the conductive element, has an important effect on the conductivity of the polymer composite proposed as an embedded element for welded joints [13].

The filler particles in the polymer matrix should form a conductive framework, which is not observed for composites with a random distribution of particles in a conventional system [14,15,16]. This is essential as the electrophysical properties of the control element are dependent on the distribution of both pure filler and also more complex hybrid carbon fillers [17,18,19]. An important aspect is the choice of polymer matrix [20,21,22], which has a pronounced effect on the ability to formulate the filler framework, as well as compatibility with the weld material. The relevant polymer material contributes to many of the required advantageous characteristics, namely, lightness, low cost of the material compared to metal, corrosion resistance, manufacturing of embedded elements of complicated geometric shapes, and ease of material processing [23]. To obtain high conductivity of the element, a high concentration of filler with random distribution is required, which leads to a deterioration in the mechanical properties of the material. Some solution of this problem can be carbon nanomaterials, e.g., carbon nanotubes or other graphene materials (graphene nanoplatelets, fullerenes, graphitic nanofibers, etc.). However, carbon black, as a predominantly amorphous carbon material, in contrast to graphene nanomaterials with predominantly sp^2^ hybridization, shows greater thermal resistance and dimensional stability, primarily in terms of degradation of both the edges and the basic graphene surface in nanomaterials. For laser welding techniques in particular, this is visible as a side effect, i.e., carbon nanotube walls with some defects can be observed just after 6 s irradiation [24]. Additionally, there is a significant tendency to agglomeration [25], which weakens larger weld areas during thermal changes in welding. CNTs and other graphene materials are partially degraded during arc welding. Evidence of poor wetting of graphene materials by most commercial polymers [26] and degradation during arc welding process were found in many studies [27]. However, the use of graphene nanomaterials like carbon nanotubes and graphene nanoplatelets as an additional component along with typical carbon black-type fillers can be justified to improve electrothermal properties, which is planned as a continuation of this research.

In fusion welding, an integrated heating element, typically made of materials like CPCs, is positioned between two polymer parts to be joined. Depending on the needs of the technology, this operation is carried out at the polymer melting point using either direct current or alternating current. Direct current is preferable in fusion welding when it comes to polymer composite elements because it provides better control over the heating temperature, which leads to a more uniform and stable melting of the polymer materials. Thus, the quality of the weld is improved. Fusion welding is the industry standard for attaching intricately formed plastic parts, and has the benefits of tight connections and high strength while taking less processing time than conventional welding techniques [28,29].

The heating element has to generate sufficient heat during welding to melt both itself and the adjacent polymer layer of the parts to be joined. To achieve this, significant overheating is required. An increase in the carbon black concentration of the composite to 30 vol.% (30CB/HDPE) has the potential to increase the heat release of the heating element and, therefore, result in a stable welded joint [29]. The resistance of the sample is approximately 8 ohms, compared to 89 ohms for a filler content of 12 vol.% (12CB/HDPE), indicating that the power received by the heating element is almost an order of magnitude higher.

The development of simple and rapid methods of forming heating elements for high-performance electrofusion welding was the goal of Kolisnyk et al. [29]. Another important and practical purpose of the study performed in this area is to use a conductive heating element that remains in the welding zone as a damage sensor sensitive to changes occurring in the welded joint. This type of sensor can allow continuous nondestructive testing of welded joints. Additionally, the application of embedded heating elements allows combinations in further industrial designs of welding and strain sensors in the final welded joint. 

The most common method of strain measurement (in real-time monitoring) is the use of electrical strain gauges. Depending on the material and economical efficiency, both fiber optic strain sensors and conventional strain sensors can be used. All types of sensors have the potential to be used individually or as part of an integrated sensor network, especially in applications with polymer materials. Carbon nanomaterials with various structures, such as graphene oxide, reduced graphene oxide, carbon nanotubes, and carbon black [30,31,32,33], effectively distributed within the polymer matrix are promising candidates for piezoresistive and pyroresistive strain gauges. 

This work presents a new method for the formation of polymer–carbon composite elements for the characterization and continuous monitoring of the quality of welded joints. The composite elements are based on a combination of HDPE as a polymeric material and an electrically conductive carbon filler of different volume content. The present work aimed to study the pyroresistive and sensing properties of composite materials based on high-density polyethylene and carbon fillers, which form a segregated system in a polymer matrix volume as an alternative to the usual solutions. They also serve as strain sensors for quality control of welded joints during their work lifetime. 

## 2. Materials and Methods

### 2.1. Materials

Electrically conductive polymer composite samples with segregated (ordered) structures were formed by the hot compaction method [34,35]. The polymer matrix of composite material was powdered high-density polyethylene (HDPE) with density ρ = 0.95 g/cm^3^, melting temperature T = 120 °C, and a particle size of 100–300 μm. Carbon black (ENSACO^®^ 250 G) with a density of ρ = 1.8 g/cm^3^ was used as the filler. 

### 2.2. Preparation of CB/HDPE Composites

The homogeneous mixture of fine HDPE powders and fillers was obtained by thorough mechanical mixing in a porcelain mortar. As a result, the conductive layers of filler particles on the surface of the polymer grains on the polyethylene surface were formed. The prepared mixture was loaded into a steel mold, preheated to a temperature above the polymer melting point (for HDPE the temperature was T = 145 °C), and then was placed under a hydraulic press and kept under a pressure of 20 MPa for 10 min with intensified air cooling until complete cooling. The formed filler pattern on the surface of the polymer granules remained when they were pressed into a mold and stuck together into a continuous polymer matrix, creating a framework (segregated structure) of the conductive phase. A diagram for the process preparation of a sample with a segregated structure is given in ref. [36]. Composite samples with a diameter of 30 mm and a thickness in the range of 1.4–1.5 mm were used for electrical conductivity measurements of as-prepared samples and after pyroresistive heating. During the composite preparation, three different volumetric amounts of carbon black were used (20, 25, and 30%).

### 2.3. Measurement Techniques

X-ray diffraction (XRD) studies of composite samples were performed using the D8 Advance diffractometer (Bruker, Karlsruhe, Germany) equipped with a Cu–Kα cathode (λ = 1.54 Å) operating at 40 kV and 40 mA. The scan rate was established at 0.60°/min with scanning step 0.02° in a range of 10° to 120° 2Θ. Measurement data collection was performed using the LYNXEYE XE-T linear detector (Bruker AXS, Karlsruhe, Germany) [37]. Identification of fitted crystalline phases was carried out using the ICDD PDF#2 database connected with the DIFFRAC.EVA program. The crystallinity of HDPE composites was calculated using the peak decomposition method [37,38]. 

Scanning electron microscopy (SEM) studies of carbon black powder and CB/HDPE composites at different stages were performed using Quanta FEG 250 (ThermoFischer Scientific, Waltham, MA, USA) by using low vacuum mode and 10 kV acceleration voltage. The initial carbon black particles were studied as deposited on carbon tape, while for CB/HDPE composite samples, after initial and secondary pressing, the area of fractures from tensile tests were studied. 

Raman spectra of CB powder superimposed on aluminum foil were collected using WitecAlpha M300+ (WITec GmbH, Ulm, Germany) equipped with Nd:YAG laser with an excitation wavelength of 532 nm and a laser power of 50 mW. The measurement parameters were as follows: laser power—1 mW, time of exposure—5 s, number of scans—50, and an acquisition range of 0–3800 cm^−1^. For each sample, approximately 7–10 points were analyzed to obtain insight into the sample’s structural homogeneity by using the spectrometer live mode without recording the spectra. The ratios of the intensities and areas of the D and G peaks (D/G), estimating the ordering of the carbon material, were determined using Witec Project 4.1 software.

The bulk electrical conductivity of the conductive polymer composites, both in the initial state and after deformation, was measured using a Keithley 6485 picoampermeter (Keithley Instruments, Cleveland, OH, USA) associated with LabVIEW 13.0 software. As electrodes, the high-purity silver paste was placed on both sides of the samples. Subsequently, copper wires were attached to electrodes. An initial voltage of 0.1 V was applied using an external power supply. The samples were kept for 300 s under the specified voltage to guarantee more stable initial conductivity values.

A universal testing machine (Instron 4204, Instron, Norwood, MA, USA) was used to evaluate the tension properties. The tensile samples were cut from as-pressed disc to obtain desired shape (4 mm × 30 mm) and subsequently tested at a crosshead speed of 2 mm/min, according to the PN-EN ISO527-1/2 procedure [39]. Based on mechanical strength results, the elastic deformation for CB/HDPE composites was limited by a strain value of 0.33%. 

The sensory responses of composites were determined under tensile deformations. Measurement tests were carried out on a DEBEN Micro tensile machine with a 2 kN traverse and dedicated DEBEN 6.3.40 software [38]. The traverse speed was set to 0.4 mm/min, and the working distance between the clamps was positioned at 15 mm. Changes in current were registered in real-time using Keithely 6485 picoammeter. According to the mechanical characteristics of the CB/HDPE composites described in [40], the elastic deformation corresponds to a strain of about 0.33%. Therefore, two different variants of tensile tests were performed, on the elastic deformation only (the strain of 0.33%) and considering plastic deformation of the electroactive composite (up to break). All tests were performed at least on three different samples in order to obtain reliable results. 

## 3. Results and Discussion

### 3.1. Surface Morphology Study of CB/HDPE Composites

**Figure 1:**
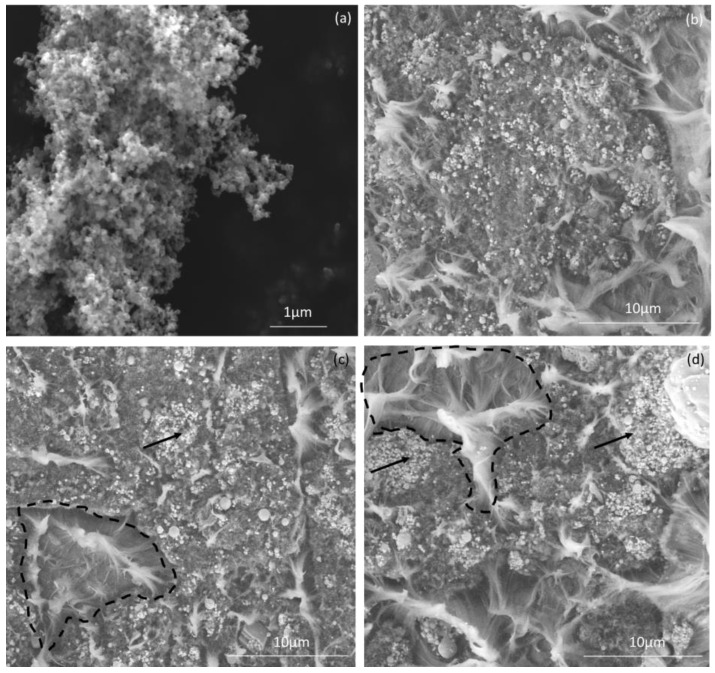
SEM micrographs of carbon black filler (**a**) and the fracture surface after tensile tests of composites with 20% vol. CB (**b**); 25% vol. CB (**c**); and 30% vol. CB (**d**). Arrows indicates CB-rich regions, while HDPE-rich regions are marked with dashed lines.

The SEM microphotographs of the initial carbon black filler and CB/HDPE composites with three different carbon black contents (20, 25, and 30%) are shown in Figure 1. 

As shown in Figure 1a, carbon black particles are varied in shape and size, and are typically composed of small (10 to 80 nm in diameter), single, sphere-shaped carbon black particles, depending on the method of preparation and precursor, and large (10 to 50 μm) chains and clusters of these particles that have formed agglomerates or aggregates of carbon black (greater than 100 μm), as was observed on an SEM microphotograph (Figure 1a).

These particles are suitable for effective reinforcement in polymer matrices due to their high specific surface area and finely partitioned particles. When uniformly distributed, nanostructured carbon black particles contribute to the composite materials’ remarkable mechanical properties such as increased modulus and strength [40]. Moreover, it is easier to ensure good-quality contact with the polymer matrix, which ultimately improves load transfer and interfacial adhesion. Furthermore, carbon black is an excellent material for applications that require improved electrical or thermal properties due to its high electrical conductivity and thermal stability [40]. Overall, the introduction of carbon black as a composite filler offers significant improvements in material performance and structural integrity, making it fundamental in the development of advanced polymer composites.

The SEM images of CB/HDPE composites reveal a segregated polymer/carbon system, with pure HDPE areas covered by high-density layers of CB nanoparticles. The CB nanoparticles homogeneously cover the HDPE surface and are found as small, densely packed spheres with diameters of tens to hundreds of nanometers. Some large agglomerates of carbon black are observed as well.

This microstructural arrangement is an indication of the segregation process, in which mechanical blending facilitates the formation of well-defined polymer–carbon boundary interfaces. As a consequence, carbon black nanoparticles electrostatically adhere to the surface of HDPE particles, forming continuous conductive layers and, after hot pressing, a conductive network within the polymer matrix is obtained, thereby enhancing the electrical properties of final carbon–polymer composites. 

The resulting segregation allows for precise controlled distribution of the filler, facilitating the tailoring of material properties to specific application requirements, especially in the case of the process of plastic welding. This involves optimization of electrical conductivity, mechanical strength, and thermal stability. Furthermore, segregation reduces the percolation threshold, as an indicator of the filler concentration required to achieve continuous conductivity pathways. This phenomenon is particularly positive for applications requiring improved conductivity at lower filler loadings [41]. 

The segregated distribution of the filler material results in a structure within the polymer matrix with characteristics proportional to the polymer particle size. In Figure 1d, for the composite with the highest content of carbon black particles, pronounced dark areas surrounding the polymer matrix can be seen compared to the SEM images of the other two composites (20 and 25 vol.% CB).

The SEM images of CB/HDPE composites furthermore show a complicated configuration of deformed high-density polyethylene fragments interspersed with carbon black filler, with the fracture surface providing the obvious presence of a crazing process coming from multiple sites. The study of the fracture surface allows us to conclude that the crazing process occurs with the crack stopping during the interrupted fibril formation in the fracture zone, depending on the amount of carbon black in the composite.

### 3.2. Structural Analysis of CB/HDPE Composites

**Figure 2:**
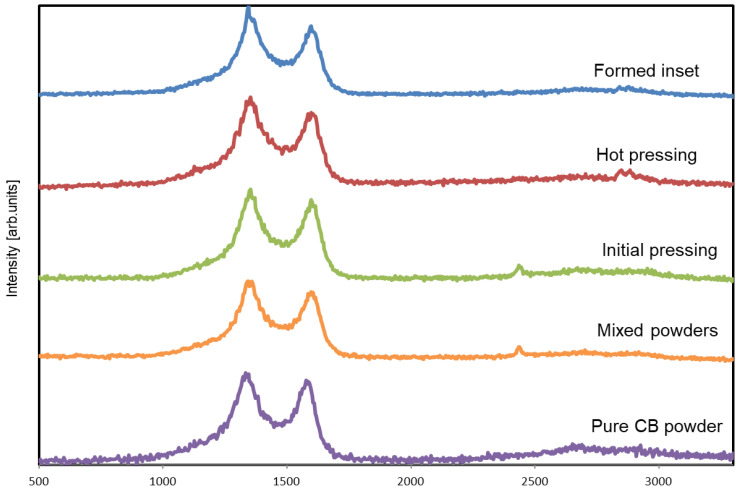
Raman spectra of HDPE-based composite with 30 vol.% of carbon black at different processing stages.

Figure 2 shows the Raman spectra of the CB powder, initial mixture of CB and HDPE grains, and CB/HDPE composites at different processing stages. The spectra of all studied materials show characteristic bands for sp^2^ and sp^3^ carbon structures [42]. The application of Raman spectroscopy allows us to determine the character of the transformation of carbon structures during CB/HDPE composite processing.

Figure 2 compares the Raman spectra of the composite filled with 30 vol.% carbon black in four different states: initial HDPE grains covered with CB particles; after initial pressing; after secondary hot pressing; formed composite insert for molded joints referenced to the pure CB. All spectra show three characteristic Raman bands that prove the presence of the carbon material in composite samples, such as the D band with intensity at 1345–1353 cm^−1^, G band at 1594–1600 cm^−1^, and 2D peaks at approximately 2700 cm^−1^. The D band corresponds to the vibration of the sp^3^-hybridized atoms of carbon related to the defects in the carbon structure, and the G band corresponds to the in-plane vibration of the sp^2^-hybridized atoms of carbon and the 2D band. In addition, the 2D band is a D-band overtone. The I_D_/I_G_ ratio is similar for all samples, about 0.85, which indicates the nonexistence of transformation of carbon structures during composite processing. as shown in Table 1. Additionally, Raman shifts at 1060, 1135, and 1300 cm^−1^ corresponding to the C–C stretch and twisting of the –CH_2_ groups in the HDPE can overlap with a stronger signal of the D-band of the carbon material because of its low intensity [11]. The other bands with Raman shifts at ~2850 cm^−1^, related to the asymmetric stretching in the crystalline and amorphous phases, and at ~2900 cm^−1^, related to the symmetric stretching in the amorphous and crystalline phases of HDPE, can be concealed in the Raman spectrum. 

**Figure 3:**
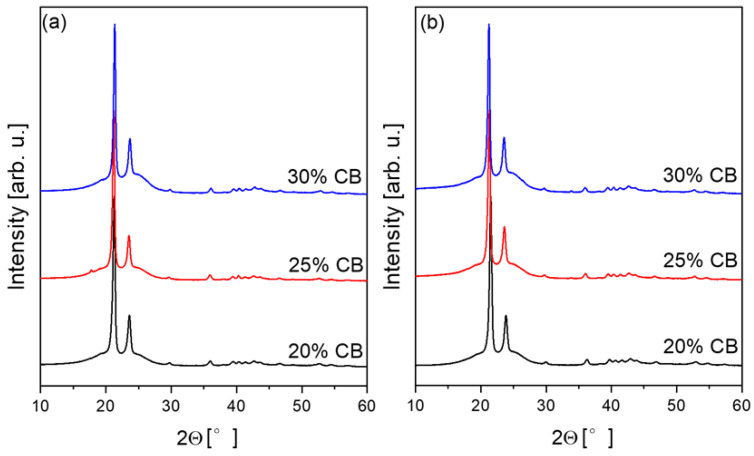
XRD patterns of CB/HDPE composites: (**a**) initial samples after hot pressing, (**b**) samples after pyroresistive heating.

Figure 3 presents XRD patterns of the CB/HDPE composites before and after pyroresistive heating, while Table 2 lists calculated values of polymer matrix crystallinity It should be noted that all peaks were identified as an orthorhombic HDPE (PDF#00-060-0986), with only a small amount of monoclinic phase (PDF#00-054-1981). Moreover, after pyroresistive heating, the traces of the monoclinic phase were detected, due to the reduction in peak intensity at 19.4° and 25.1° 2Θ. The pyroresistive heating of the composites up to the temperature of about 250 °C results in the decrease in polyethylene crystallinity. The relative change in crystallinity is about 6% for 25CB/HDPE and 30CB/HDPE, while for 20CB/HDPE it is 3.5%. Rapid cooling and resulting residual stress can lead to uneven stress distribution, affecting the crystalline and amorphous phases of HDPE differently.

### 3.3. Electrical and Heating Properties of CB/HDPE Composites

The well-known and widely used percolation model (Equation (1)) was applied to describe the experimental data of the electrical conductivity in a wide CB concentration range for the CB/HDPE composites:

(1) $$\sigma =\sigma_{m}{\left(\varphi -\varphi_{c}\right)}^{t}$$

where *φ* is the filler concentration, *φ_c_* is the percolation threshold value, the parameter *σ_m_* is related to the filler conductivity value, and *t* is the critical exponent. Fitting Equation (1) to a linear relationship, log *σ*~log (*φ* − *φ_c_*), gives the possibility to find the value of the critical exponent *t* and the filler conductivity *σ_m_*. 

**Figure 4:**
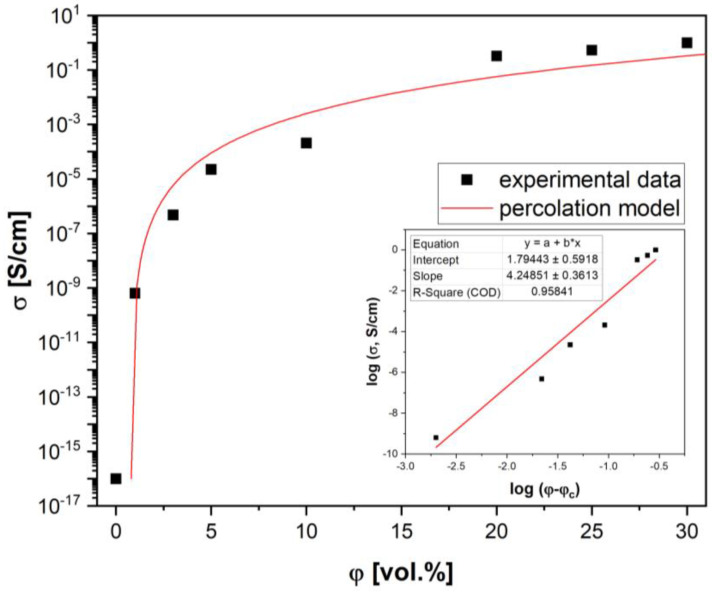
Percolation curve for composites CB/HDPE. The inset shows the linearization of the percolation curve in log coordinates *σ*~log (*φ* − *φ_c_*).

The results presented in Figure 4 confirm that this model is in good agreement with the experimental data with the following parameters: *φ_c_* = 0.8 vol.%, *σ_m_* = 61.7 S/cm, and *t* = 4.25, with matching linearity fit probability *R*^2^ = 0.9584. 

The values of percolation threshold *φ_c_* and critical exponent *t* differ from statistical values due to the nonrandom distribution of the filler in the polymer matrix.

A lattice model proposed by Mamunya et al. [43] was used to evaluate the properties of the segregated system:

(2) $$\frac{\varphi }{\varphi_{loc}}=1-{\left(1-\frac{nd}{D}\right)}^{3}$$

where *φ* is the average filler concentration in the composite volume, *φ_loc_* is the local filler concentration within the segregated structure, *n* is the number of filler particle layers in the intergranular region, and *D* and *d* are the diameters of polymer and filler particles, respectively.

To obtain the insulator–conductor transition concentration (*φ* = *φ_c_*), the assumption is that HDPE particles have a diameter of *D* = 150 μm, CB has particles of *d* = 0.5 μm, the required number of layers of filler particles equals *n* = 2, and the local filler concentration should be in the range of 0.36 to 0.43 [44]. In this case, the theoretical value of *φ_c_* = 0.79 vol.%, and is in good agreement with the experimental one. 

The obtained critical exponent *t,* meanwhile, is much higher than the theoretical value 2, predicted for a random distribution of the filler [45,46]. The ordered distribution of the conductive phase in segregated composites leads to an increase in *t* [36,47]. The wall thicknesses of 35, 50, and 67.5 μm for concentrations in the range of 20, 25, and 30 vol.%, respectively, were calculated from Equation (2).

Further work [19,21,22,33] showed the influence of the positive temperature coefficient (PTC) on the pyroresistive properties of composites based on semicrystalline and amorphous polymers. The relationship achieved was pronounced for concentrations slightly above the percolation but does not describe the behavior of highly filled composites. 

**Figure 5:**
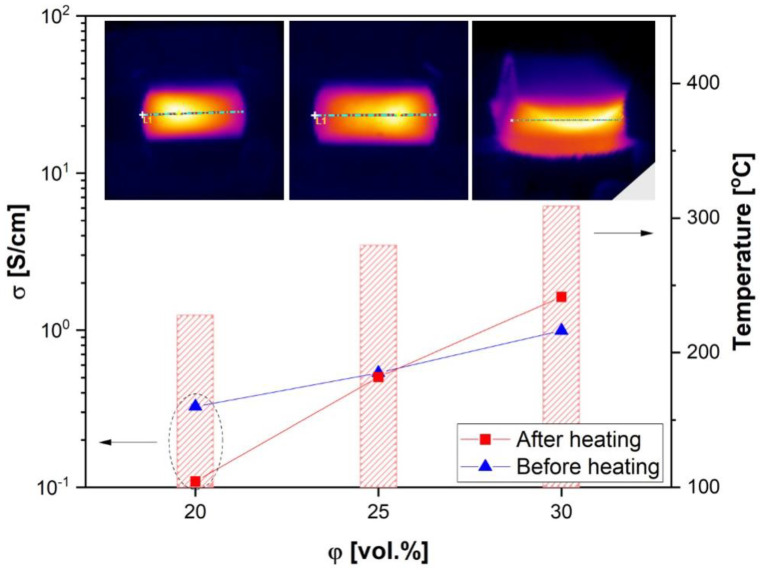
Electrical conductivity before (blue points) and after (red points) pyroresistive heating of segregated composites CB/HDPE; bars correspond to temperatures reached during the heating process. The inserts (IR images) show the temperature distribution for the selected sample at any given concentration.

The effect of pyroresistive heating on electrical conductivity is shown in Figure 5. The heating process has been shown not to change the properties dramatically, and the slight variation for 20 and 30 vol.% CB can be explained by the dominance of filler properties in the overall composite, i.e., the effect of PTC decreases as the amount of filler increases. Additionally, the inserts in Figure 5 show a good temperature distribution during heating, which is consistent with the concept of highly filled composites in which the filler particles are in direct contact and create a conductive network. 

During heating in a segregated system, the thermal expansion of the polymer is expected, i.e., the dimensions of the lattice elements increase. According to the previously discussed model [47], some parameters of the segregated composites can be calculated: for the filler concentration of 20 vol.% and polymer particle size of *D* = 150 μm, the thickness of the intergranular layer is *nd* = 35 μm, the size of the unfilled part of the polymer is *L* = 115 μm, and, consequently, the *L/nd* ratio = 3.3. An increase in concentration leads to a decrease in *L*/*nd* ratio, i.e., the thickness of the intergranular layer increases, and the size of the unfilled area of the polymer decreases. For the segregated system with a filler concentration of 20 vol.%, the size of the unfilled area *L* is much larger than the thickness of the intergranular layer *nd*. When heated, the thermal expansion of the polymer significantly affects the packing of filler particles in the intergranular layer and contributes to the destruction of the conductive paths between the filler particles and a decrease in the electrical conductivity of the composite. Increasing the filler concentration leads to a decrease in the size of the unfilled area of the polymer and a decrease in its effect on network deformation and electrical conductivity. At the highest filler concentration (30 vol.%), the lattice thickness, namely, the intergranular layer *nd*, becomes comparable to the size of unfilled areas *L*, and thermal expansion leads to additional packing of the intergranular layer and a slight increase in conductivity (Figure 5).

Using the measured temperature values, it is possible to calculate the thermal energy required to heat the samples using the following equation:

(3) Q=C·M·ΔT

where *C* is the specific heat capacity of the composite calculated according to the mixture rule, *M* is the mass of the composite, and ∆*T* is the temperature change.

The Joule heat generated by electricity can be calculated:

(4) $$A=\frac{U^{2}}{R}\cdot t$$

where *U* is applied to the composite voltage during time *t*, and *R* is the resistance of the composite. 

For composites studied, the values calculated from Equations (3) and (4) are listed in Table 3.

The A/Q ratio obtained in this study (approx. 1.5) is significantly lower than previously obtained values (6.7) [47]. This can be explained by the noticeably dissimilar filling degree of the composites in the study presented (20–30 vol.% versus 8 vol.% in [47]).

### 3.4. Sensory Properties of CB/HDPE Composites

**Figure 6:**
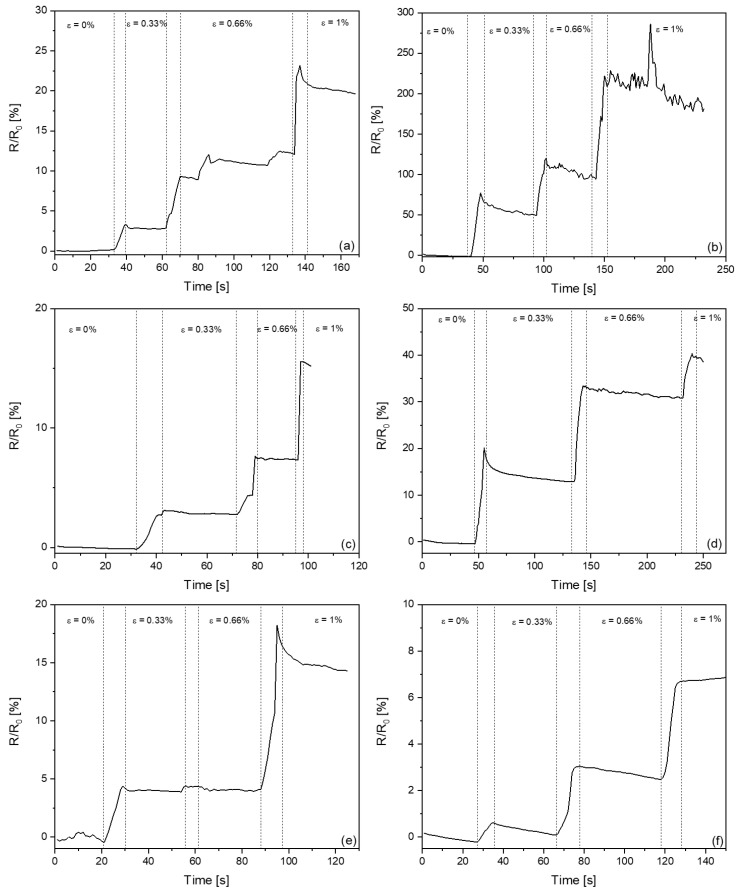
Changes in resistance observed during tensile tests for composites with various carbon black content: (**a**) 20CB/HDPE, (**b**) 20CB/HDPE PH, (**c**) 25CB/HDPE, (**d**) 25CB/HDPE PH, (**e**) 30CB/HDPE, and (**f**) 30CB/HDPE PH. Subscript PH in samples’ description refers to composite after pyroresistive heating.

Figure 6 shows the sensory responses of the composites as a function of time, and Table S1 summarizes the sensory responses of HDPE-based composites with different amounts of carbon black and after different processing steps. It was found that all the initial materials tested responded to all applied deformations (from 0.33% to 1%), but the intensity of the response varied. All initial samples showed similar responses to 0.33% strain (in the range of 2.5 to 3.4% initial resistance and conductivity), but it should be noted that this is in the elastic deformation range for HDPE-based composites (see Table S1 and Figure S1). The relative change in resistance and conductivity for 20CB/HDPE, 25CB/HDPE, and 30CB/HDPE were 2.42/−2.45, 2.76/−2.62, and 3.07/−3.37, respectively. Plastic deformation (0.66% and 1% strains) showed a further increase in responses (Figure 6). The strongest responses were obtained for the composite with the lowest conductivity (20CB/HDPE, relative change in resistance and conductivity 19.21 and −16.21, respectively), while the weakest for the composite with the highest conductivity (30CB/HDPE, relative change in resistance and conductivity 14.19 and −12.73, respectively). It should also be noted that different stresses were detected for different samples (see Table S1). The higher the amount of carbon black in the composite, the higher the stresses detected for the same deformation. 

The electroactive composites after pyroresistive heating showed significantly different behavior to the untreated ones (Figure 6). For composites with 20 vol.% CB, the initial resistance increased compared to the initial samples, which improved the sensory properties. For the composite with 30 vol.% carbon black, the resistance decreased, resulting in a significantly lower sensitivity of the composite. The best responses were detected for the composite containing 20 vol.% CB, due to the highest initial resistance (50.7 Ω, see Table S1), suggesting intense disruption of the conductive pathway during pyroresistive heating. The relative change in resistance and conductivity for the 0.33% strain was 51.08 and −34.56, respectively, compared to 2.42/−2.45 obtained for the untreated sample. This indicates a significant improvement in the sensitivity of the heated composite, especially in terms of elastic deformation. The plastic deformation of the 20CB/HDPE composite after pyroresistive heating showed a further increase in resistance and decrease in conductivity, as the polymer matrix flowed. 

The composite containing 25 vol.% carbon black after pyroresistive heating also showed significantly higher changes in resistance and conductivity than the untreated sample (4 times higher for elastic deformation, 2.5 times higher for plastic deformation; see Figure 6 and Table S1), but the final resistance (at 1% deformation) was below 25 Ω, which is significantly lower than the initial resistance of 20CB/HDPE after pyroresistive heating. This makes it possible to use the composites as an embedded sensor for strain monitoring, operating at low voltages.

After pyroresistive heating, the 30CB/HDPE composite showed deteriorated sensory properties compared to the initial one. As mentioned earlier, this is most likely due to the very high amount of carbon black particles in the polymer matrix, which can create new contacts during pyroresistive heating. In the case of elastic deformation, the response was more than 10 times lower than that detected for the initial sample, suggesting that these composites cannot be used as an embedded sensor for strain monitoring. It should also be noted that the composites after pyroresistive heating showed lower stresses for the same strain than the initial composites.

In the literature, the use of piezoresistive and sensory materials for structural condition monitoring of laminated structures [48,49] and resin-based composites [50,51] has been mainly described. In epoxy-based composites, the average change in resistance is between 1 and 1.5%, as described by Spinelli et al. [51] for 0.3% MWCNT/epoxy and by Wang et al. [50] for 3% of CNT-GNP/epoxy mixture. Although the two teams used different carbon nanomaterials and their quantities, the results obtained for elastic deformation were very similar. It should be mentioned that the CB/HDPE composites presented here showed higher sensitivity than the epoxy-based composites reported in the literature [50,51]. 

On the other hand, Shen et al. [48] described the potential for detecting the glass-fiber-reinforced polymer (GFRP) delamination using carbon nanotube bucky papers. Their results showed a strong dependence on the orientation of glass fiber fabrics, with a change in resistance ranging from 40 to 2000%. Wang et al. [49] showed the potential of CNT- and RGO-coated carbon fibers in glass fabric to detect laminate deformation. The effect depended on the coating used on the carbon fiber, with resistance changes ranging from 0.7 to 6% at laminate break. On the other hand, the flexible materials were highly explored [52,53,54]. Jang et al. [52] described a highly flexible composite with excellent sensitivity (up to 160%) using CNTs and carbonyl iron powder as a reinforcement. Reports on elastomer [52,53,54] and thermoplastic [40,55] piezoresistive sensors can also be found in the literature. However, neither elastomer nor thermoplastic composites have been used as embedded strain sensors so far. 

Therefore, here, we presented the idea of applying a thermoplastic composite with carbon reinforcement as a heating element during electrofusion welding, and its application in structural health monitoring of welded joints shows great potential application. However, further studies on actual localization in welded joints must be performed.

## 4. Conclusions

This work presents, for the first time, the potential of electroactive HDPE-based composites with carbon black for use as embedded heating elements and strain sensors in electrofusion-welded joints. The effect of CB quantities on the pyroresistive and sensory properties of the composites was evaluated and described. The effect of self-heating on the composite structure and sensory properties was demonstrated. The main conclusions can be drawn as follows: 

The pyroresistive properties of CB/HDPE composites with 20%, 25%, and 30% of CB were studied and are similar and allow electrofusion welding on polyethylene components. This welding procedure is expected to leave an electroactive insert within the welded joint, which can be further used as an embedded strain sensor.

The pyroresistive heating procedure, similar to the normal electrofusion welding procedure, affects the structure of the polymer matrix, reducing its crystallinity and partially disrupting the conductive pathways. This affects not only the conductivity of the composites but also their mechanical strength. 

All initial composites show good sensory properties, but after pyroresistive heating, composites with 20 and 30 vol.% carbon black exhibit dissimilar behaviors. The 20CB/HDPE shows significantly higher resistance, which can be measured with a simple device, while the 30CB/HDPE shows the poorer sensory properties of all the composites studied. These composites cannot be used as an embedded sensor for strain monitoring due to too-high or too-low responses for different deformations.

The composite with 25 vol.% CB has the best balance between pyroresistive properties as well as sensory properties after heating. Therefore, further studies will be performed to investigate this composite as an embedded strain sensor in welded joints.

## Figures and Tables

**Table 1:** Raman shift signals from the CB/HDPE.

	D (cm^−1^)	G (cm^−1^)	*I*_D_/*I*_G_	*A*_D_/*A*_G_
Pure CB powder	1329	1573	0.84	1.95
Initial CB/HDPE mixture	1345	1600	0.84	1.89
After initial pressing	1353	1598	0.85	1.75
After hot pressing	1353	1598	0.85	1.98
Formed insertion part	1345	1594	0.84	1.95

**Table 2:** Crystallinity degree of CB/HDPE composites determined using X-ray diffraction.

CB Amount (vol.%)	Crystallinity of HDPE Matrix (%)	
	Initial Samples	After Pyroresistive Heating
20	54	51
25	54	48
30	53	46

**Table 3:** Parameters of Equations (3) and (4).

φ (vol.%)	Q (J)	A (J)	A/Q
20	177.2	269.2	1.519
25	223.4	339.6	1.520
30	248.8	378.5	1.521

## Data Availability

The raw data supporting the conclusions of this article will be made available by the authors on request.

## Supplementary Materials

The following supporting information can be downloaded at: https://www.mdpi.com/article/10.3390/ma17153776/s1, Table S1. Mechanical and sensory properties of HDPE/CB composites in initial state and after pyroresistive heating. Figure S1. Stress-strain curves typically obtained for CB/HDPE with various amounts of CB.
