# Supplementary material for: Heating and Strain Sensing Elements Based on Segregated Polyethylene/Carbon Black Composites in Polymer Welded Joints
Source: Materials (Basel). 2024 Aug 1;17(15):3776. doi: 10.3390/ma17153776 (PMC11312915; doi:10.3390/ma17153776)
Supplement: Supplementary file 1 [file materials-17-03776-s001.zip › materials-3086229-supplementary.pdf]

SUPPLEMENTARY INFORMATIONS

Table S1. Mechanical and sensory properties of HDPE/CB composites in initial state and after pyroresistive heating.

	20CB/HDPE		25CB/HDPE		30CB/HDPE	
	**Initial**	**Pyroresistive heated**	**Initial**	**Pyroresistive heated**	**Initial**	**Pyroresistive heated**
**Tensile strength**, MPa	11.4 (8)	-	13.9 (7)	-	18.5 (5)	-
**Elastic deformation**, %	0.64 (1)	-	0.54 (03)	-	0.60 (03)	-
**Initial resistance, Ω**	17.477 (116)	50.701 (116)	15.584 (116)	16.943 (116)	12.947 (116)	9.716 (116)
**Initial conductivity,** S/cm	0.327 (010)	0.109 (010)	0.535 (006)	0.504 (006)	0.994 (010)	1.630 (020)
**Resistance after deformation of about 0.33%, Ω**	17.900 (119)	76.597 (123)	16.015 (121)	19.207 (112)	13.344 (099)	9.735 (127)
**Change of resistance for strain 0.33%,** %	2.420	51.077	2.766	13.362	3.066	0.196
**Conductivity after deformation of about 0.33%,** S/cm	0.319 (003)	0.071 (022)	0.521 (021)	0.445 (018)	0.960 (010)	1.626 (050)
**Change of conductivity for strain 0.33%,** %	-2.446	-34.562	-2.617	-11.706	-3.372	-0.245
**Detected stress,** MPa	1.576	1.079	2.250	1.750	3.625	3.500
**Resistance after deformation of about 0.66%, Ω**	19.461 (119)	100.235 (099)	16.726 (123)	22.259 (119)	13.409 (123)	9.976 (119)
**Change of resistance for strain 0.66%,** %	11.352	97.700	7.328	31.376	3.568	2.676
**Conductivity after deformation of about 0.66%,** S/cm	0.294 (011)	0.054 (007)	0.498 (022)	0.384 (019)	0.956 (010)	1.587 (010)
**Change of conductivity for strain 0.66%,** %	-10.092	-50.230	-6.916	-23.810	-3.775	-2.638
**Detected stress,** MPa	2.540	2.182	3.583	3.500	5.813	5.167
**Resistance after deformation of about 1%, Ω**	20.835 (099)	171.674 (119)	17.984 (119)	23.461 (123)	14.785 (119)	10.394 (121)
**Change of resistance for strain 1%,** %	19.214	238.604	15.400	38.470	14.196	6.978
**Conductivity after deformation of about 1%, Ω**	0.274 (012)	0.032 (003)	0.463 (022)	0.364 (017)	0.867 (010)	1.523 (010)
**Change of conductivity for strain 1%,** %	-16.208	-70.507	-13.458	-27.778	-12.733	-6.564
**Detected stress,** MPa	3.556	2.909	4.250	3.667	6.563	5.667

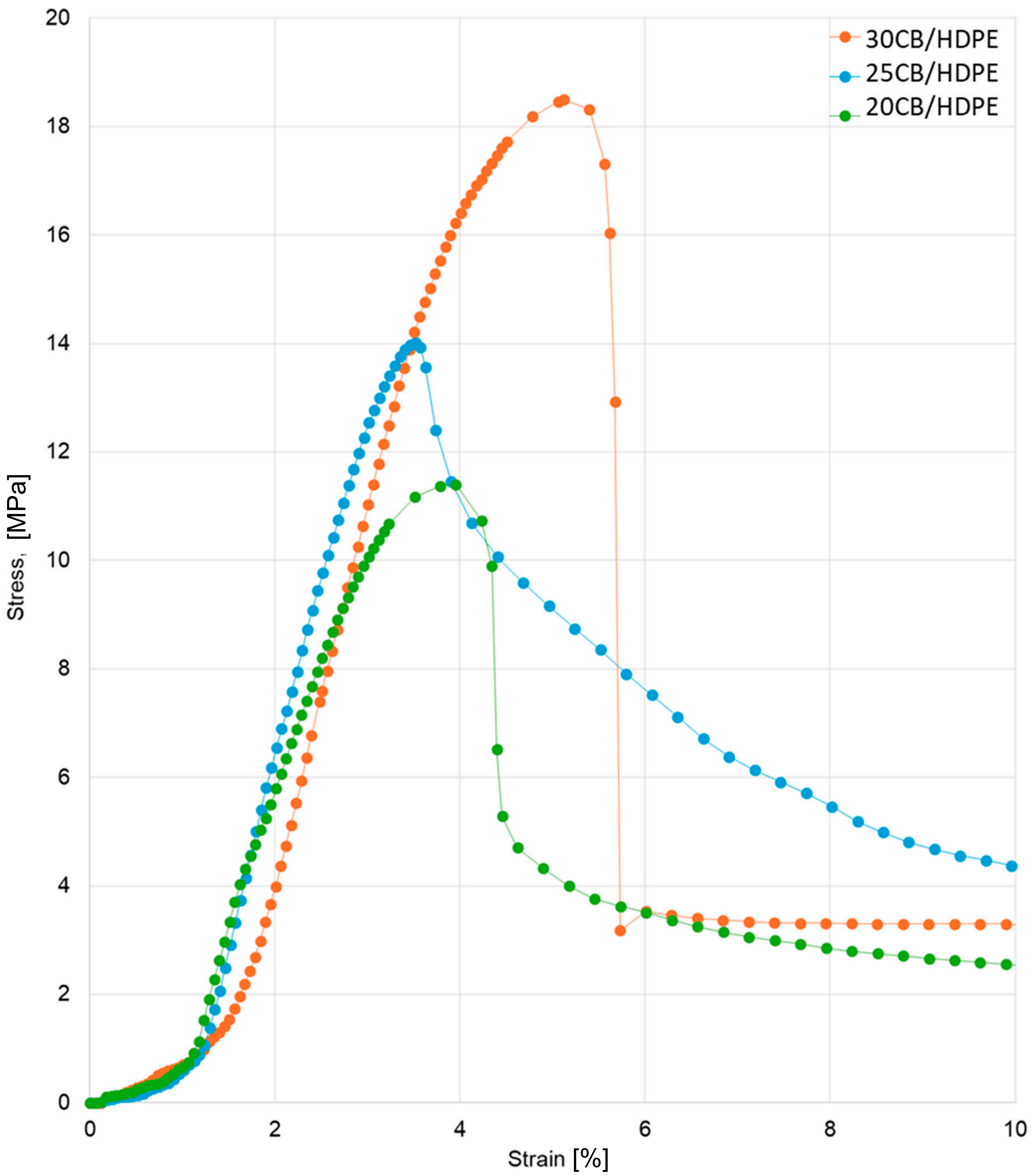

Figure S1. Stress-strain curves typically obtained for CB/HDPE with various amounts of CB.
